# Regulating embryo models in the UK

**DOI:** 10.1093/jlb/lsae016

**Published:** 2024-07-18

**Authors:** Emily Jackson

**Affiliations:** Law School, London School of Economics and Political Science, London, WC2A 2AE, UK

**Keywords:** embryo research, stem cell based embryo models, embryo models, embryoids, regulation, induced pluripotent stem cells

## Abstract

One of this century’s most dramatic scientific developments is the reprogramming of stem cells in order to create self-organizing embryo-like entities, known as stem cell based embryo models (SCBEMs). The science is moving very quickly, but if, as increasingly appears to be the case, scientists are capable of creating entities that are effectively indistinguishable from sperm and egg derived embryos, important legal questions arise. In countries like the UK, where a strict regulatory regime applies to research on embryos, should this be extended to SCBEM research, or would a different regulatory response be appropriate? Drawing on the 1984 Warnock Report, the Human Fertilisation and Embryology Act 1990 and the latest guidelines from the International Society for Stem Cell Research, this article considers principles for the regulation of the creation and use of SCBEMs.

## I. INTRODUCTION

One of this century’s most dramatic scientific developments is the reprogramming of stem cells in order to create 3D models of organs and tissues. This can be done with human embryonic stem (hES) cells, but more commonly involves the use of induced pluripotent stem (iPS) cells. Often derived from skin cells, these are cells that have been reprogrammed into an embryonic-like pluripotent state. As Eisenstein has put it:

Inside every stem cell is an organ waiting to happen—biologists have known this for generations. But only recently have they learned how readily that potential can be unlocked in culture.^1^

Twice in the last 5 years—organoids in 2018,^2^ and ‘models for modelling development’ in 2023^3^—this technology has been chosen as *Nature Methods*’ ‘method of the year’.

As well using iPS cells to create organoids,^4^ and in vitro derived gametes (sperm and eggs),^5^ it is also possible to create embryo-like entities, which the International Society for Stem Cell Research (ISSCR) describes as stem cell based embryo models (SCBEMs).^6^ In its 2021 guidelines, the ISSCR explained that ‘[a]dvances in cellular engineering make possible the assembly, differentiation, aggregation, or re-association of cell populations in a manner that models or recapitulates key stages of embryonic development’.^7^ The science is moving very quickly, but if, as increasingly appears to be the case, scientists are capable of creating entities that are effectively indistinguishable from embryos that were created from sperm and eggs, important legal questions arise. In countries like the UK, where a strict regulatory regime applies to research on embryos, should this be extended to SCBEM research, or would a different regulatory response be appropriate?

In this article, as well as looking forwards, to explore how the UK should regulate the creation and use of SCBEMs, I will also look backwards, to the 1984 report of the *Committee of Enquiry into Human Fertilisation and Embryology*, chaired by Mary Warnock (Warnock Committee). Set up in 1982, 4 years after the birth of Louise Brown, the first baby to be born as a result of in vitro fertilization (IVF), the Warnock Committee was established in order ‘to examine the social, ethical and legal implications of recent, and potential developments in the field of human assisted reproduction’ and to make recommendations.^8^ It is at least arguable that being able to make embryo-like entities in vitro is as dramatic a development as IVF was more than four decades ago. While some of the key questions raised by SCBEMs are different from the issues with which the Warnock Committee grappled in the early 1980s, I will suggest that its approach to the regulation of scientific and clinical innovation continues to be relevant and helpful 40 years later.

## II. WHAT ARE SCBEMS AND WHAT IS THE PURPOSE OF THIS RESEARCH?

Although their complexity exists on a spectrum, the ISSCR has distinguished between two different types of SCBEMs. Non-integrated SCBEMs ‘experimentally recapitulate some, but not all aspects of the peri-implantation embryo’, and they ‘do not have any reasonable expectations of specifying additional cell types that would result in formation of an integrated embryo model’.^9^ In contrast, integrated SCBEMs ‘contain the relevant embryonic and extra-embryonic structures and could potentially achieve the complexity where they might realistically manifest the ability to undergo further integrated development if cultured for additional time in vitro’.^10^ It is thought that ‘at some point of refinement, embryo models could pass a “Turing test”, meaning that an evaluator testing them without having information about their origin could not distinguish them from embryos’.^11^ At the risk of crude over-simplification, some integrated SCBEMs might *theoretically* be capable of implanting in a uterus, whereas non-integrated SCBEMs could not.

Scientists’ interest in creating SCBEMs is not just in order to test the limits of what can be done with stem cells. Rather, the purpose of this research is to increase understanding of early embryogenesis, including the processes of implantation and gastrulation, which until now have occurred ‘hidden inside the womb’.^12^ Lack of knowledge about early embryological development means that we do not know why as many as 70 per cent of embryos fail to result in a live birth.^13^ Most of these losses occur during the first 6 weeks of development, with 50 per cent occurring before implantation.^14^ The loss of a preimplantation embryo may be imperceptible, but between 12 and 24 per cent of established pregnancies end in miscarriage.^15^ Although IVF success rates have improved in recent years,^16^ failure continues to be the most likely outcome for every embryo transfer.^17^ Chromosomal and congenital abnormalities—which are a major cause of prenatal death and childhood morbidity and mortality—are also poorly understood.^18^

Scientists can use donated (or created) human embryos in research, but embryo models have three distinct advantages over IVF embryos. First, they can be manufactured at scale: it would be possible to make hundreds of thousands of SCBEMs relatively easily, whereas donated embryos are a scarce and precious resource. Combined with gene editing, SCBEMs can be tweaked to ‘be used in high-throughput genetic tests and drug screens — procedures that generally form the basis of therapeutic discoveries’.^19^ Secondly, SCBEMs can be standardized and controlled, using gene editing, in a way that donated embryos cannot.^20^

Thirdly, and this depends upon the answer to a question considered in the next section, if embryo models are not subject to the same regulatory regime as IVF embryos, it would be possible to carry out research into embryogenesis at a later stage of development. It is a criminal offense under section 3 of the Human Fertilisation and Embryology Act 1990 to culture an embryo in vitro for more than 14 days. Gastrulation (when cells start to differentiate into organs) takes place in what is often referred to as the ‘black box’ of embryological development,^21^ between 14 and 28 days. It would be illegal to carry out research on embryos after 14 days, and, for practical reasons, between 14 and 28 days embryos cannot be observed in vivo via ultrasound, and nor can they be donated by women undergoing terminations of pregnancy.^22^ Gastrulation is critical to the embryo’s development: in a *Horizon* program in 1986, Lewis Wolpert fleshed out a quotation which had been attributed to him (and embellished in its repetition):

It’s not birth, death or marriage which is the most important event in your life, but gastrulation; and it is a very important event in early development, because you get enormous changes in the location of cells in the early embryo, so they get put in the right place so that organs can then really begin to develop.^23^

It would therefore be hard to overstate the significance of being able—for the first time—to observe gastrulation in vitro.

## III. WHAT IS THE LEGAL STATUS OF AN INTEGRATED SCBEM?

If it is possible to make integrated SCBEMs that are effectively indistinguishable from IVF embryos,^24^ what is their legal status? If they are ‘embryos’ for the purposes of the Human Fertilisation and Embryology Act 1990, they would be subject to the same strict limits as research on human embryos. Unsurprisingly, given that it was updated most recently in 2008, the Act is silent on the question of whether it applies to embryo models. Instead, section 1(1) rather circuitously states that: ‘In this Act … embryo means a live human embryo and does not include a human admixed embryo (as defined by section 4A(6))’.^25^

There is no further definition of the word ‘embryo’ in the Act, but section 1(1) would be an unusual ‘definition’, since it contains the word it is ‘defining’ (‘embryo means a … embryo’). A better interpretation, according to the House of Lords in *R (on the application of Quintavalle) v Secretary of State for Health* (*Quintavalle*),^26^ is that ‘embryo’ should be given its ordinary language meaning, and the point of section 1(1) is not to define what an embryo *is*, but instead to specify which *types* of embryo—that is, live and human—are subject to regulation.

According to the Oxford English Dictionary, an ‘embryo’ is an ‘unborn human offspring, esp. during the early stages of development’, while the Oxford Dictionary of Biology states that an embryo is:

An animal in the earliest stages of its development, from the time when the fertilized ovum starts to divide, while it is contained within the egg or reproductive organs of the mother, until hatching or birth. A human embryo is called a fetus after the first eight weeks of pregnancy.

SCBEMs are not ‘unborn human offspring’, and nor do they result from a fertilized ovum. If we were to ask whether scientists consider SCBEMs to be embryos, it would appear that most do not,^27^ although some have argued that the definition of embryo should change in order to include SCBEMs.^28^ At the time of writing, the Human Fertilisation and Embryology Authority (HFEA) is working on the assumption that SCBEMs are *not* embryos for the purposes of the Act, and are therefore not subject to its regulatory regime.^29^

This assumption could, of course, be superseded by a decision of the UK Supreme Court, if it were to rule that SCBEMs are embryos for the purposes of the Act. This happened two decades ago, when the House of Lords in *Quintavalle* decided that embryos created by cell nuclear replacement (CNR) (the method used to clone Dolly the sheep) were embryos. It did this by ‘construing new techniques in a way that incorporates them into the special regulatory framework’.^30^ A primary consideration, as Lord Steyn explained, was that:

Parliament intended the protective regulatory system in connection with human embryos to be comprehensive. This protective purpose was plainly not intended to be tied to the particular way in which an embryo might be created. The overriding ethical case for protection was general.^31^

But even if an overriding imperative of the Act was that there ‘was to be no free for all’,^32^ in 2003 there was no doubt that CNR embryos were embryos. In contrast, it would strain the rules of statutory interpretation to expand the application of an Act that regulates research on live, human embryos so that it also applies to 3D models.

Although it has not yet been used, the Secretary of State does have the power to expand what counts as an embryo through Regulations. Under section 1(6) of the Act:

If it appears to the Secretary of State necessary or desirable to do so in the light of developments in science or medicine, regulations may provide that in this Act (except in section 4A) ‘embryo’, ‘eggs’, ‘sperm’ or ‘gametes’ includes things specified in the regulations which would not otherwise fall within the definition.

Introducing Regulations which provided that embryo models should come within the definition of ‘embryo’ for the purposes of the Act would be a relatively straightforward way to subject SCBEM research to regulation, but the Act does not permit the HFEA to impose a lighter touch regulatory regime on certain types of embryo. Instead, if the section 1(6) power were to be used, it would have an immediately chilling effect on SCBEM research in the UK. All the scientists who are currently working in this field would have to apply for licenses from the HFEA before their work could continue, and they would have to comply with the strict licensing requirements set out in the Act and the HFEA’s Code of Practice.

Currently, in the absence of such Regulations, SCBEMs are not subject to any special regulatory regime in the UK. Unlike embryos, there are therefore no time limits on how long SCBEMs can be cultured in vitro, nor are there any developmental landmarks that must not be passed. There are no restrictions on the purposes for which SCBEM research can be carried out, and scientists do not need to be inspected or licensed by the HFEA before they are allowed to carry out this sort of research.

In its recommendations to government on reform of the 1990 Act, the HFEA has said that it may ‘be proportionate to amend the Act to permit some form of statutory regulation of stem cell-based embryo models in the future’.^33^ New primary legislation will take time, however, and the need for governance arrangements is sufficiently pressing that, in the meantime, scientists are working with other stakeholders to come up with an interim code of practice,^34^ in the same way as a Voluntary and then Interim Licensing Authority preceded the establishment of the HFEA in the 1980s.^35^ A further consequence of the slow and laborious legislative process is that, if new legislation does eventually come before parliament, it will be important to try to ‘future-proof’ its provisions, perhaps by enabling as yet unforeseen scientific developments to be accommodated through ‘soft law’ or guidance.^36^

## IV. WHAT IS THE MORAL STATUS OF AN INTEGRATED SCBEM?

If scientists can create a new sort of entity which is very like an embryo, but is not an embryo, what is that entity’s moral status, or, to put it another way, what are our moral obligations toward it? In order to answer this question, it might be helpful to identify exactly what features or qualities of a human embryo give rise to its moral status. If what matters is that an embryo results from the fertilization of a human egg by human sperm, and/or that (in almost every case) IVF embryos were created as part of a ‘parental project’, SCBEMs would have a different moral status. But if it is the intrinsic form and capacities of an embryo that are critical, it will be necessary to work out whether SCBEMs could ever be essentially the same thing.

Debates over the moral status of an embryo often focus on the significance of its ‘potential’. It is, for example, sometimes claimed that the embryo’s potential to become a person gives it a special moral status, albeit falling short of full personhood.^37^ In contrast, there are those who believe that because the embryo is continuous with the person it might become, it has the same moral status as that person.^38^ Others counter that having the potential to become something does not mean we need to treat an entity as if it already were that something.^39^

Of course, in practice no IVF embryo has the potential to spontaneously become a person. The IVF embryo’s potential depends entirely upon whether it is transferred to a uterus and successfully gestated for at least 5 months.^40^ Moreover, the special treatment of embryos under the Human Fertilisation and Embryology Act 1990 does not vary according to whether an embryo is viable. Embryos that are non-viable, and that could never have any potential to become a person, are nevertheless subject to the same regulatory regime as viable embryos. It is therefore not the potential of individual embryos to become persons which gives them a special status in law, because this exists only for a subset of viable, implanted embryos.

Induced pluripotency further disrupts arguments grounded in potential, because, as Piotrowska explains, it means that:

nearly any cell in a human body has the potential to develop into a human being. Given this observation, how are we supposed to develop the idea that only certain cells have the unique potential to develop into human beings, thereby justifying the idea that they should be afforded special oversight?^41^

It would be hard to argue that all skin cells (and all the other cells that might be used in iPSC research, like blood) deserve special protection in the same way as an early human embryo.

In addition, SCBEMs give rise to a more specific and practical difficulty, because we simply do not know whether an SCBEM could have the potential to become a person. The experiments that would be necessary to answer this question would pose wholly unacceptable risks to the health of women and their offspring, and would therefore be unethical, now and for the foreseeable future (although, as we see below, they would not be illegal in the UK). The potential of an SCBEM is therefore unknown and, for the time being, unknowable.

At this point, it is instructive to revisit the Warnock Committee’s approach to the question of the moral status of the embryo. Its report acknowledged that there were radically different views in society, and among Committee members, on when life and personhood began. These were not ‘questions of fact, susceptible of straightforward answers’, but were instead ‘complex amalgams of factual and moral judgements’.^42^ Consensus on such polarized and polarizing questions was never going to be feasible. It was, however, possible to reach agreement that some legislation would be better than none:

There must be some barriers that are not to be crossed, some limits fixed, beyond which people must not be allowed to go. Nor is such a wish for containment a mere whim or fancy. The very existence of morality depends on it. A society which had no inhibiting limits, especially in the areas with which we have been concerned, questions of birth and death, of the setting up of families, and the valuing of human life, would be a society without moral scruples. And this nobody wants.^43^

The important question for the Warnock Committee was therefore not what the embryo *was*, but *how* it should be treated, and what limits should be placed upon its use in order to secure public trust and confidence.^44^

Importantly, Warnock recognized that regulation was not only necessary in order to address public concerns. It was also positively helpful for scientists to have clear boundaries set down in advance by parliament.^45^ In reflecting upon her Committee’s work, Warnock explained that:

everyone wants legislation: the general public so that they can be certain that no nameless horrors are going on, hidden away in laboratories; the scientific community so that they may be in a position to get on with their work, without the threat of private prosecutions, or disruption by those who object to what they are doing. Many scientists also want the onus of deciding what is and what is not morally acceptable to be partially lifted from their shoulders.^46^

Applying the Warnock approach to SCBEMs, the question becomes what restrictions upon the creation and use of SCBEMs will provide reassurance to the public and clarity for scientists?

## V. PUBLIC ENGAGEMENT

As well as deftly sidestepping the irresolvable question of the moral status of the embryo, the Warnock Committee was ahead of its time in acknowledging that it matters how the public feels about controversial scientific developments.^47^ The importance of genuine two-way dialog between scientists and the public was later reasserted in the 2000 House of Lords report, *Science and Society*, published in response to the collapse of trust in science that followed the introduction of genetically modified foods without public consultation.^48^ There is now widespread agreement that, before deciding how SCBEMs should be regulated, there should be extensive public engagement and dialog about what matters to people and why.^49^

In 1984, one of the most important and difficult questions for the Warnock Committee was whether embryo research should be allowed at all.^50^ In the face of fundamental moral disagreement, the priority for the Warnock Committee was having trustworthy regulation in place, with the precise content of the rules mattering less than the overriding imperative of there being some boundaries rather than none.^51^ This is consistent with evidence from public engagement that having some ‘red lines’ in place is seen as the best way to deter and control ‘rogue scientists’, or those who might be tempted to ‘go too far’ as a result of commercial or funding pressures.^52^

He Jiankui’s premature clinical use of CRISPR/Cas9 to genetically modify embryos,^53^ and Woo Suk Hwang’s fraudulent claims to have been the first to clone a human embryo and derive stem cell lines from it,^54^ are invoked as cautionary tales of what happens in the absence of effective regulation.^55^ In their interview study, Bates et al. found that members of the public often expressed skepticism over whether it was possible to be sure that unethical practices were not happening anyway.^56^ They also found that independent regulators, like the HFEA, were more trusted to prevent unethical practices than self-regulation and peer review. An independent regulatory regime may therefore promote public trust in and of itself, regardless of exactly what substantive limits are set down by law.

The Human Developmental Biology Initiative, funded by Wellcome and UKRI Sciencewise, has recently published the results of its public dialog workshops about the future regulation of embryo research.^57^ Although its main focus was the 14 day limit for embryo research, participants were also asked about the regulation of SCBEMs, and most were clear that regulation was necessary. Some participants pointed to their ‘human essence’, in order to argue that SCBEMs should be regulated in the same way as embryos, while others thought that they were research tools and that a different regulatory system would be appropriate. Participants hoped that models could be used instead of embryos, in order to save this precious and finite resource for research that can only be done on embryos. Concerns were also expressed about perfectionism, and the use of this sort of research to eradicate disabilities and reduce diversity.^58^ More public dialog workshops specifically on embryo models are taking place early in 2024.

While there is a consensus that genuine public engagement involves two-way dialog with the public about what matters to them,^59^ rather than adopting the now-discredited ‘deficit’ model of public education, it is also important to acknowledge that public understanding of SCBEM (and embryo) research is low. Public engagement therefore requires scientists to be able to explain clearly and accessibly what this research involves and what it hopes to achieve.

As we have seen, the main driver of SCBEM research is to understand more about embryogenesis, with the hope that this knowledge could be put to clinical use in the future in order to reduce miscarriage rates, improve IVF success rates, and reduce the incidence of congenital disease.^60^ SCBEMs could also speed up the process of drug discovery, and facilitate better understanding of medicines’ teratogenic effects, that is, their tendency to cause abnormalities following fetal exposure during pregnancy.

It is, however, important that justifications for this sort of research do not ‘overpromise’,^61^ and create ‘false hope’ that a cure for miscarriage or dementia is imminent.^62^ It is also important that scientists do not ‘underpromise’ about the developmental potential of SCBEMs: the public might be reassured by declarations that SCBEMs could never result in a pregnancy, but it is important to be honest that this is a question to which it is impossible to give a definitive answer.^63^

## VI. REGULATING SCBEMS

When thinking about how SCBEM research should be regulated in the UK, two existing sets of rules and guidance might offer a useful starting point. First, aspects of the Human Fertilisation and Embryology Act 1990 could be adapted in order to encompass SCBEM research. For example, in line with the statutory limits on embryo research, it might be important to set a time limit or biological marker beyond which SCBEMs should not be allowed to develop, and to consider what sort of oversight is appropriate, and how tough the penalties should be for non-compliance.

Secondly, the ISSCR’s 2021 guidance sets out ‘an internationally coordinated framework to regulate … human stem cell research, clinical translation, and related research activities’.^64^ Although the guidelines do not have the force of law, ‘they complement existing legal frameworks and can inform the interpretation and development of laws applicable to stem cell research as well as provide guidance for research practices not covered by legislation’.^65^

### VI.A. Identifying a Regulator

According to the ISSCR, different levels of oversight are required for non-integrated SCBEMs and integrated SCBEMs: non-integrated SCBEMs are merely ‘reportable, but not typically reviewed by a specialist oversight process’, while integrated SCBEMs should be ‘reviewed by a specialist oversight process’. Transferring SCBEMs to a woman’s uterus falls in the ISSCR’s ‘not allowed category’.^66^

Putting the ISSCR guidance into practice in the UK would require decisions to be taken about which body should be carrying out the ‘specialized oversight’ of integrated SCBEMs, and to whom scientists should be reporting research on non-integrated SCBEMs. The ISSCR itself suggests that in the UK, the ‘HFEA and regional ethics committees (RECs), are well positioned to perform review and oversight of embryo and related research’.^67^

It is easy to see why the HFEA might be identified as the appropriate reviewer of research on integrated SCBEMs in the UK. The HFEA has more than three decades’ experience of the regulation of research on embryos, during which time it has had to deal with a wide range of new questions, including the licensing of CNR research and research on human admixed hybrids. It would also be possible for the HFEA to be the body to whom research on non-integrated SCBEMs should be reported.

Giving the HFEA regulatory authority over SCBEM research would expand the HFEA’s remit, and would require new primary legislation, and additional funding. In recent years, the HFEA’s primary focus has been the protection of patients undergoing fertility treatment, and it would be important to ensure that a significant expansion of its role in the regulation of research did not dilute or divert attention away from its focus on the interests of patients.

For those entities which should be subject to full specialized review – described by the ISSCR as ‘review, approval, and ongoing monitoring’ – as well as identifying which body might be charged with these tasks, it is also necessary to decide what criteria it should be applying when deciding whether to approve a project. The ISSCR specifies that it should consider ‘the scientific rationale and merit of research proposals, the relevant expertise of the researchers, and the ethical permissibility and justification for the research’. It is relatively straightforward to judge a proposal’s scientific merit and researchers’ expertise, but more complex is the question of how to draw a distinction between ethically permissible and ethically impermissible research on integrated SCBEMs.

One practical difficulty is the distinction the ISSCR draws between integrated and non-integrated SCBEMs. As more is understood about SCBEMs’ complexity and their capacity to self-organize, enforcing and policing a bright line boundary between different types of SCBEM will not be straightforward. Aside from whether the boundary itself will continue to make sense, ensuring that integrated SCBEMs have not been wrongly categorized as non-integrated would pose an additional burden on the regulator.^68^ Perhaps, rather than laying down in law different regulatory approaches to different types of SCBEM, which are themselves likely to be superseded by scientific developments, it would be more sensible to give the regulator discretion over the limits it imposes on all SCBEM research, which could be tailored to the ethical complexity of different types of model.

### VI.B. No Clinical Use

One obvious initial red line would be a prohibition on the clinical use of SCBEMs in fertility treatment. Indeed, section 3(2)(a) of the Human Fertilisation and Embryology Act 1990, as amended, attempts to prevent the transfer into a woman’s body of anything other than a ‘permitted embryo’ (created by the fertilization of an egg ‘from the ovaries of a woman’ by sperm ‘from the testes of a man’). However, it does so through what turns out to be an unfortunate choice of words: ‘No person shall place in a woman *an embryo* other than a permitted embryo’ (emphasis added). As a result, if (as is currently assumed to the case) an SCBEM is not an embryo for the purposes of the Act, it could lawfully be transferred to a woman’s uterus.

The fact that it would not be unlawful to transfer an SCBEM to a uterus does not mean it is likely to happen in the UK, however. Given the danger this would pose to the woman’s health, as well as the wholly unknowable risks to the entity that might develop within her body, transferring an SCBEM to a uterus would be unsafe and unethical, and represent a breach of a clinician’s professional obligations. There is a consensus among clinicians and scientists that there should be no clinical use of SCBEMs for the foreseeable future. If SCBEMs were to be regulated, it nevertheless seems unarguable that this inadvertent legislative gap ought to be plugged, and that any new legislation should prohibit placing an embryo model in a woman’s body.

A further consideration is that the SCBEM will be a clone of the person whose cell was used to create the iPS cell (or if hES cells were used, a clone of the embryo from which the hESC line was derived).^69^ It is, therefore, interesting to consider whether another reason for ruling out SCBEM’s clinical use might be revulsion or squeamishness at the prospect of human cloning, and the fear that – in the wrong hands – SCBEMs could be implanted in order to produce multiple human clones.^70^ If the use of SCBEMs in fertility treatment were ever to become plausibly safe in the future, it may be necessary to return to debates that were common 25 years ago, in the aftermath of Dolly the sheep’s birth, about whether human reproductive cloning could ever be ethically acceptable.

### VI.C. A Prohibition on the Placing of a Human SCBEM in an Animal

Section 3(3)(b) of the Human Fertilisation and Embryology Act 1990 makes it a criminal offense to place a human embryo in an animal. Given the potential dangers involved in placing a human SCBEM in an animal host, it would be important to replicate this prohibition for SCBEMs.

### VI.D. A Time or Developmental Limit?

Under section 3 of the Human Fertilisation and Embryology Act, it is a criminal offense to culture embryos in vitro after the appearance of the primitive streak^71^ or after 14 days, whichever happens first. While the primitive streak/14 day limit has played a critical role in the UK’s regulatory regime,^72^ and has been widely copied worldwide,^73^ it does not work for embryo models. Unlike embryos, there is no equivalent to fertilization as a ‘day zero’ for SCBEMs. SCBEMs also do not develop in a linear way ‘from the one-cell stage through progressive steps of complexity’, and can instead ‘jump in … to a later stage (Day 17) without developing the primitive streak’.^74^ Pointing to evidence that in SCBEMs ‘the emergence of [morally significant] features can be suppressed (or hastened)’, Piotrowska points out that

a rule that uses number of days or the appearance of the PS [primitive streak] as relevant markers is not going to reliably track what we take to be morally salient. The rule has come untethered from the features it was meant to protect and consequently should be replaced.^75^

Even if the 14 day ‘deadline model’ does not work for SCBEMs,^76^ some limit upon how far SCBEMs might be allowed to develop in vitro might be required. In practice, there may be a point at which further development becomes impossible, given the absence of signals from a maternal uterine environment. Nevertheless, permitting SCBEMs to develop indefinitely would almost certainly be unacceptable. In mice, embryo models have been cultured until they developed ‘headfolds with defined forebrain and midbrain regions and … a beating heart-like structure’.^77^ And as Pereira Daoud et al. put it:

regardless of whether or not [SBCEMs] qualify as human embryos, they may still develop features that many would consider morally concerning, such as incipient brain activity or an emerging human form.^78^

The 2021 ISSCR guidelines recommend that integrated embryo models should be cultivated ‘for the minimum time necessary to achieve the scientific objective’.^79^ In advocating a flexible and open-ended time limit, the ISSCR is treating SCBEMs differently from IVF embryos, for which they argue that culture beyond 14 days should be contemplated only if there is ‘broad public support’, backed up by ‘local policies and regulations’. Whether this sort of goal-based limit would be sufficient to promote public confidence is as yet unknown, but the risk is that it looks like a limit that could be extended again and again, as scientific objectives change. Perhaps this sort of limit could be accompanied by another limit, expressed as an embryological developmental stage (perhaps the emergence of the first neural structures), or as a number of days. Embryo models could therefore be cultured for the minimum time necessary to achieve the scientific objective, or until X occurs, or for no more than 28 days, whichever is the sooner. More permissive still would be a limit tied to the capacity to experience pain (which even on the most cautious estimate would not be until around 20 weeks^80^).

Whatever limit is chosen, there may be a parallel between embryos/SCBEMs and the distinction that was drawn in the 1980s between the ‘embryo proper’ and the ‘pre-embryo’.^81^ The ‘embryo proper’ was said to exist when the primitive streak appeared at around 14 days, after which twinning is no longer possible. Before that, the ‘pre-embryo’, as Warnock herself put it, ‘hasn’t decided how many people it is going to be’.^82^ The idea that the ‘pre-embryo’ might have a different status from that of the ‘embryo proper’ was summed up in the words of Anne McLaren (the Warnock Committee’s influential developmental biologist, to whom the 14 day rule has been attributed): ‘If I had to point to a stage and say “This is when I began being me”, I would think it would have to be here”’.^83^ More cynically, it has been suggested that the concept of the ‘pre-embryo’ was employed in the 1980s in order to bolster support for permissive legislation,^84^ and that it was abandoned after it had served its purpose.^85^

In the same way as the terms ‘pre-embryo’ and ‘embryo proper’ were used in order to distinguish between those entities that could and those that could not be used in research, the term SCBEM might continue to be valuable—even if an SCBEM were to pass a ‘Turing test’ and be indistinguishable from an embryo—in order to subject it to different rules and red lines from those which apply to IVF embryos.

### VI.E. Statutory Purposes

In relation to embryos, a research license ‘cannot authorise any activity unless the activity appears to the Authority to be necessary or desirable’ for one of the statutory purposes, which include increasing knowledge about serious disease and the development of embryos, promoting advances in the treatment of infertility, increasing knowledge about the causes of miscarriages, and developing more efficient techniques of contraception.^86^ It would not be possible to obtain a license to use human embryos in drug toxicology testing. As we have seen, the creation of SCBEMs at scale would enable medicines to be tested for teratogenic effects,^87^ and since hardly any medicines have been tested adequately on pregnant women,^88^ this could be transformative for pregnant women’s health.

The current statutory purposes for embryo research would therefore be too restrictive for SCBEM research, if it were to fulfil its potential to speed up and refine drug discovery. Indeed, it may be unnecessary to specify that only certain purposes would be legitimate, and instead ethical review could simply consider whether the research was justifiable in the public interest.

### VI.F. The Necessity Principle or a Preference for Less Complex Models?

Research on embryos is further limited by the ‘necessity principle’. Schedule 2(3)(5) of the Act requires the HFEA’s License Committee to refuse a license for research on embryos if scientists could carry out the research without using embryos, for example by using animal models or existing stem cell lines. As a result, under the current law, if a proposed research project could use SCBEMs instead of embryos, it would not be possible to obtain a license to use embryos in research.

Applying a ‘necessity principle’ to SCBEM research would not make sense. SCBEMs are non-sentient and can be manufactured at scale, and thus have the potential to replace both animal models and embryos. Constraining the use of SCBEMs by requiring scientists to prove that there would be no other way to do their research would potentially hinder valuable research—which could also minimize harm to sentient animals—without any obvious benefits.

More plausible would be a principle that scientists should not make models that are more complex and complete than they need to be in order to carry out their research project. If a model is ‘perfect’, some might question whether it still makes sense to call it a model, and whether it has instead become the thing itself,^89^ which in the case of embryos would make them subject to a very strict regulatory regime. As Daoud et al. put it, the ‘paradox that emerges here is that the better these models become, the less useful they may be’ as replacements for embryos, if there ‘is a tipping point beyond which greater similarity collapses into identity’.^90^

If an SCBEM is not identical to an embryo, it may be easier to treat it as a simple research tool, rather than as an embryo, or an entity occupying a complex liminal status.^91^ As a result, some leading stem cell scientists have argued that it would be better to engineer SCBEMs to be less ‘complete’. For example, Rivron et al argue that:

when pursuing a particular goal, models that are less entitled to protection should be preferred. Forming an embryo model that is more complete than necessary might provide the same benefit but raise more concerns. Therefore, if possible, less complete models should be preferred.^92^

While Hyun has expressed his ‘hope’ that

various organoid models focused on isolated developmental events, together with purposefully incomplete gastruloid models, will provide, in the aggregate, a beautifully unified portrait of human development such that singular, more biologically complete but morally confusing human models will not be necessary.^93^

Of course, if an incomplete model can serve the same purpose as a complete one, it will often be simpler and easier to do the research on an incomplete model. But if a complete model would be more useful for research purposes, should there be a preference for less useful research tools, because they raise fewer ethical dilemmas? Or might it possible to strike a balance through which:

embryo-like models should be as similar as possible to human embryos in order to support their utility as a research substitute, while remaining sufficiently different to preserve distinctions that ethically permit research.^94^

Whether it is necessary to preserve differences between a stem cell-based model and the ‘real thing’ in order to enable research to continue will depends upon how acute one regards the ethical dilemmas which arise from the creation of SCBEMs. On one hand, if they are so serious that they cast doubt on whether the research should proceed at all, it may be better to opt for less complete models. If, on the other hand, the ethical dilemmas are considered manageable within a strict regulatory regime, then—within those limits—it makes sense to strive to produce the most useful models possible. Avoiding having to use incomplete models when complete models would be more useful, as a way to resolve ethical dilemmas, might therefore represent a further argument for regulation.

In addition, it could be argued that the complexity of an SCBEM is not a reason to abandon research, but rather that it requires more reflection and deliberation than research on a simpler model. Barnhart and Dierickx advocate a ‘moral principle of complexity’, such that the ‘more complex the organoid-entity the researchers plan to develop, the more they ought to spend in moral reflection, anticipation, and deliberation’.^95^

It is, however, worth reminding ourselves that the completeness or complexity of a model does not matter for its own sake. Rather, these qualities are significant because they make the entity more like something—a human embryo—which should be treated differently from inanimate human tissue. ‘Completeness’ does not rule out research on SCBEMs, but instead begs the question of whether this research should be subject to more restrictions than other research on iPS cells.

### VI.G. Consent

The 1990 Act requires donors of embryos to consent to their use in a specific research project,^96^ rather than being able to give ‘broad consent’ for their embryos to be banked for use in the future. In practice, this means that many IVF patients who want to donate their leftover embryos to research are unable to do so, and in its recommendations to government, the HFEA has proposed that future legislation should facilitate embryo banking.^97^ Given that project-specific consent is regarded as too restrictive for research on embryos, it would be illogical to require it for SCBEM research.

It is also important to remember that donors to SCBEM research will normally have donated skin cells to iPS research. Even if people are likely to be less attached to a skin cell than they are to an embryo which could have been their child’s sibling,^98^ some donors may nevertheless wish to be able to exercise some control over what is done with any iPS cells derived from their donation: for example, they might want to rule out the creation of gametes or animal/human chimeras.^99^ Given that an embryo model would be the skin cell donor’s clone, it is even possible that some people might find the donation of skin cells to stem cell research more unsettling than embryo donation. It would be simplest to accept donations only from people who are willing to give wholly unrestricted consent, though it might be important to ensure that they have been given some indication of the very broad range of uses to which any iPS cell line derived from their skin cells could potentially be put.

### VI.H. Penalties

Finally, another important feature of the UK’s regulatory regime for embryo research is that there are serious criminal penalties for non-compliance.^100^ A scientist who cultures an embryo in vitro for more than 14 days could go to prison for up to 10 years. Even if this sort of prison sentence seems draconian in comparison to sentences reported for rape or causing death by dangerous driving, backing up statutory red lines with criminal sanctions sends a clear signal about how seriously they are taken. The transfer of an SCBEM to a human or animal uterus would be sufficiently dangerous that a criminal penalty might be proportionate, but parliament will have to decide whether it would also be appropriate to criminalize other misuses of SCBEMs, such as cultivating them beyond any set time or developmental limit.

## VII. RELATIONSHIP BETWEEN RESEARCH ON SCBEMS AND OTHER TYPES OF RESEARCH

### VII.A. SCBEMs and Embryo Research

As we have seen, the statute currently contains an in-built presumption that SCBEMs must be used if they could replace the use of human embryos.^101^ Moris et al have suggested that ‘one major benefit to *in vitro* embryo models is that they could reduce the number of human embryos required for research, thus contributing toward a “human 3Rs” [replacement, reduction and refinement] approach’.^102^ This is a reference to the 3Rs approach to research on animals, first developed by Russell and Burch in 1959: Replacement (of conscious, living vertebrates by non-sentient alternatives); Reduction (in the number of animals used to obtain information); and Refinement (of procedures to reduce to suffering).^103^*If* it was thought desirable to limit the number of human embryos used in research, ‘embryo models could represent an alternative option that might be less ethically loaded’.^104^

That does not mean that research on SCBEMs could entirely replace research on embryos. In order to validate the use of SCBEMs, it will be necessary to compare them with human embryos.^105^ Indeed, in order to validate the use of SCBEMs which develop beyond the emergence of the primitive streak, there may be a compelling argument for replacing the 14 day time limit for embryo research with a new limit, perhaps of 21 or 28 days (which would require new primary legislation).^106^ It is also possible that there will be research projects where SCBEMs are not a good substitute for human embryos.

Moreover, not everyone would agree that reducing the number of embryos used in research is a desirable goal. Almost all embryos used in research would otherwise have been discarded following IVF treatment.^107^ Destroying embryos is not self-evidently preferable to carrying out potentially valuable scientific research on them, especially since IVF patients often express a wish to donate their leftover embryos to research, and sometimes go to considerable lengths to do so.^108^ Would-be embryo donors are often parents whose children would not have existed without embryo research, who have an understandable desire to ‘give something back’.^109^ Using donated embryos in research is therefore not just beneficial for science, but is also often the best way to respect patients’ wishes about the disposal of their embryos.

Although in a minority, some people believe that an embryo has the same moral status as a person, and that its destruction—through being used in a research project or as an inevitable consequence of IVF treatment—is equivalent to murder. On one hand, it might be assumed that SCBEM research would be more acceptable to people with faith-based objections to embryo research, since it does not involve experimenting on a human embryo.^110^ On the other hand, if SCBEMs were to become indistinguishable from embryos, opponents of embryo research might consider them to be ‘embryonic humans’ in the same way as IVF embryos.^111^

### VII.B. SCBEMs and Animal Research

There could also be advantages in using SCBEMs rather than animals in toxicological studies. Species-specific differences in development make animals an imperfect way to test the safety of new compounds. The 3Rs approach to animal research also limits their use in the earliest stages of drug discovery. In contrast, SCBEMs:

are likely to become valuable tools for screening assays, with particular potential in the fields of teratogenicity and drug discovery because of their potential ability to recapitulate human-specific features in a high-throughput manner.^112^

The suggestion that we should adopt the 3Rs approach to both animals and embryos raises the question of what to do when these approaches point in different directions.^113^ As we have seen, the 1990 Act’s necessity principle means that animal models should be used in preference to embryos. But the 3Rs approach requires us to replace animals with non-sentient materials, wherever possible. Embryos are non-sentient, but replacing animal models with embryos would be ruled out by statute. This unhelpful circularity may be inevitable if we take a 3Rs approach to two different entities, so it may be simpler to reserve the 3Rs approach for animals, which would lead to a presumption that SCBEMs (and other organoids) should be used in preference to conscious, sentient animals wherever possible. It would, however, too hasty to suggest that SCBEMs (and other organoids) could entirely *replace* research on animals. As Hinterberger and Bea point out, while ‘some studies can be run on organoids alone, bioscience journals commonly require in vivo proof of study claims’.^114^

## VIII. A SUMMARY: WHAT SHOULD REGULATION OF SCBEMS INVOLVE?

To conclude, it might be helpful to summarize a set of working principles or assumptions that could govern a new regulatory approach. First, it might be important to be explicit that embryo models are not embryos, but nor are they just cell lines. A different approach is needed, which is ‘more flexible than that for embryo research, but more stringent than that for research on traditional cell lines’.^115^ Secondly, it would be sensible to amend the Human Fertilisation and Embryology Act in order to rule out the transfer of an SCBEM to both a human and an animal uterus. Thirdly, it might be necessary to identify a developmental stage or time limit beyond which research on integrated SCBEMs should not be allowed to proceed. Defining exactly what this limit should be will not be straightforward, but regulation should rule out the indefinite culture of SCBEMs in vitro.

Fourthly, limiting the purposes for which SCBEM research could take place would be too restrictive, especially given the potential benefits to patients, and to sentient animals, if SCBEMs were capable of replacing animal models in toxicology testing. Fifthly, it may be important to consider whether the current presumption in the Human Fertilisation and Embryology Act 1990 that embryo models should always be used in preference to IVF embryos should be retained. On one hand, this sends a clear message that embryos are a precious resource, which should be used in research only when no other experiments could achieve the same results. On the other hand, given what we know about patients’ preferences, priority should also be given to ensuring that patients who wish to donate their leftover embryos to research have the opportunity to do so.

Sixthly, it is axiomatic that skin (and other) cell donors for stem cell research should give informed consent. This should be broad consent—where donors do not have to consent to a particular research project—but it would be necessary to decide whether it is preferable to only accept donations from informed donors who do not wish to exercise any control at all over the future use of iPS cells derived from their donation, or whether it might be important to give donors the option of ruling out certain future uses.

Seventhly, it would be important to determine what proper oversight of SCBEM research might look like. Seeking permission from a regulator before being permitted to create SCBEMs might be unnecessarily burdensome, and a more proportionate response could be a requirement to report on the creation and use of SCBEM in research, so that—unlike now—it would be possible for the public, the regulator, and parliamentarians to know more about the extent and nature of SCBEM research in the UK.

Finally, if there were a set of limits laid down either in statute and/or in a Code of Practice, there is a need to consider how these limits should be enforced. Although it would probably be excessive to require scientists to obtain a license before they create any SCBEMs, the regulator could have the power to carry out unannounced inspections and spot checks, as well as facilitating the reporting of instances of non-compliance, perhaps anonymously. There could be a range of penalties for a failure to comply with the law and the Code of Practice, in order to recognize that some breaches—such as transfer to a human or animal uterus—warrant a more serious response than others.

## IX. CONCLUSION

Throughout the world, whether and how SCBEM research is regulated depends haphazardly on the form of words used in embryo research legislation passed long before anyone had contemplated the creation of SCBEMs. Pre-SCBEM laws may therefore inadvertently either hamper or facilitate this sort of research. For example, Sweden regulates research on ‘fertilized eggs’, so SCBEM research is not subject to any of its restrictions. Some countries which prohibit all research on embryos, like Italy and Turkey, nevertheless permit SCBEM research by default.

In contrast, the Australian Research Involving Human Embryos Act 2002, as amended, defines the human embryo in a way that appears to include SCBEMs: ‘a discrete entity that has arisen from … any other process that initiates organized development of a biological entity with a human nuclear genome or altered human nuclear genome that has the potential to develop up to, or beyond, the stage at which the primitive streak appears’. As a result, Australian scientists who wish to carry out research on SCBEMs do so within a much more restrictive environment than that which exists in the UK.^116^

This jurisdictional variation in what is considered an embryo is a consequence of the time lag between scientific innovation and legal reform. Inevitably, when a piece of legislation is regulating a fast-moving area of science, unanticipated developments may place strain on statutory language, chosen without the benefit of hindsight. It is therefore to be expected that scientific innovation will result in gaps and oddities in the statutory regime. The most effective way to deal with the impossibility of anticipating these ‘known unknowns’ is to build in future-proofing options, such as the option of amending legislation via Regulations, or, more flexibly still, to give a trusted regulator—like the HFEA—broad discretion over the detail of the rules.

Even if the HFEA is the obvious candidate regulator for SCBEM research in the UK, this would significantly extend its remit and workload, at a time when it may be difficult or even impossible to persuade the Treasury to increase spending on the regulation of stem cell science. It is also worth acknowledging that setting up a regulatory system which complies with the ISSCR guidelines would impose additional constraints on UK scientists, who are currently creating and using SCBEMs without any regulatory oversight. We know from UK stem cell scientists’ involvement in the drafting of interim governance arrangements that sensible, carefully drafted regulations are likely to be welcomed by scientists, for whom knowing in advance where the boundaries lie is in practice more useful than uncertainty. While there are many important lessons we can learn from the Warnock Report as we embark on the process of deciding how to regulate the creation of novel embryo-like entities, perhaps the most important is that regulation can be an enabler of good science, rather than an impediment to it.^117^

## Footnotes

1 Michael Eisenstein, *Organoids: The Body Builders,* 15 Nat. Methods 19–23 (2018).

2 Method of the Year 2017: *Organoids*, 15 Nat. Methods 1 (2018).

3 Method of the Year 2023: *Methods for Modelling Developing*, 20 Nat. Methods 1831-2 (2023).

4 A. L. Bredenoord, H. Clevers & J. A. Knoblich, *Human Tissues in a Dish: The Research and Ethical Implications of Organoid Technology,* 355 Science eaaf9414 (2017).

5 T. F. Murphy**,***The Meaning of Synthetic Gametes for Gay and Lesbian People and Bioethics Too,* 40 J. Med. Ethics 762–5 (2014).

6 Scientists have also made what they have described as embryoids, gastruloids, and iBlastoids. See, for example, X. Liu et al., *Modelling Human Blastocysts by Reprogramming Fibroblasts into iBlastoids* 591 Nature 627–632 (2021); Giuseppe Pettinato, Xuejun Wen & Ning Zhang, *Engineering Strategies for the Formation of Embryoid Bodies from Human Pluripotent Stem Cells* 24 Stem cells dev. 1595–1609 (2015); Alfonso Martinez Arias, Yusuke Marikawa & Naomi Moris, *Gastruloids: Pluripotent Stem Cell Models of Mammalian Gastrulation and Embryo Engineering*, 488 Dev. Biol. 488 35-46 (2022).

7
International Society for Stem Cell Research, Guidelines for stem cell research and clinical translation (ISSCR, 2021), https://www.isscr.org/guidelines (accessed June 10, 2024).

8
Department of Health & Social Security, Report of the Committee of Enquiry into Human Fertilisation and Embryology (HMSO, 1984) (Warnock Report).

9 ISSCR, *supra* note 7.

10
*Id.*

11 Nicolas C. Rivron et al., *An Ethical Framework for Human Embryology with Embryo Models,* 186 Cell 3548–3557 (2023).

12 Thorsten E. Boroviak, *A Human Embryo Model Cracks Symmetry Breaking*, 29 Cell Stem Cell 869–870 (2022).

13 Michael J. Zinaman et al., *Estimates of Human Fertility and Pregnancy Loss,*65 Fertil. steril. 503–509 (1996).

14 Naomi Moris et al., *Biomedical and Societal Impacts of in Vitro Embryo Models of Mammalian Development*, 16 Stem Cell Rep. 1021–1030 (2021).

15 Kate MacWilliams et al., *Understanding the Experience of Miscarriage in the Emergency Department*, 42 J Emerg Nurs 504–512 (2016).

16 In 1991, patients aged 35–37 had a live birth rate per embryo transferred of 6 per cent; by 2021, this had increased to 33 per cent (*Fertility treatment 2021: preliminary trends and figures* (HFEA, 2023)).

17 Even among younger women (aged 18–34), the pregnancy rate per embryo transferred in 2021 was 41 per cent (*Fertility treatment 2021: preliminary trends and figures* (HFEA, 2023)).

18 Davor Jurkovic, Caroline Overton & Ruth Bender-Atik, *Diagnosis and management of first trimester miscarriage*, 346 Br. Med. J. f3676 (2013). Yingjuan Liu et al., *Global Birth Prevalence of Congenital Heart Defects 1970–2017: Updated Systematic Review and Meta-analysis of 260 Studies*, 48 Int. j. epidemiol. 455–463 (2019).

19 Nicolas Rivron et al., *Debate Ethics of Embryo Models from Stem Cells*, 564 Nature 183–185 (2018)

20 Peter J. Rugg-Gunn, Naomi Moris & Patrick P. L. Tam, *Technical Challenges of Studying Early Human Development*, 150 Development dev201797 (2023) ; Amy Hinterberger & Sara Bea, *How do Scientists Model Humanness? A Qualitative Study of Human Organoids in Biomedical Research,* 320 Soc. Sci. Med. 115676 (2023).

21 Kenichiro Taniguchi, Idse Heemskerk & Deborah L. Gumucio, *Opening the Black Box: Stem Cell–based Modeling of Human Post-implantation Development*, 218 J Cell Biol 410–421 (2019).

22 Eszter Posfai et al., *All Models Are Wrong, but Some Are Useful: Establishing Standards for Stem Cell-based Embryo Models* 16 Stem Cell Rep. 1117–1141 (2021); Jianping Fu, Aryeh Warmflash & Matthias P. Lutolf, *Stem-cell-based Embryo Models for Fundamental Research and Translation*, 20 Nat. Mater. 132–144 (2021).

23 ‘Genesis’, Horizon, BBC, first broadcast 13 Jan. 1986. For a fascinating account of the life of Wolpert’s famous ‘quotation’, see Nick Hopwood, ‘*Not Birth, Marriage or Death, but Gastrulation’: The Life of a Quotation in Biology,* 55 Br. J. Hist. Sci. 1-26 (2022).

24 Rivron et al., *supra* note 19; B. Oldak et al., *Complete Human Day 14 Post-implantation Embryo Models from Naïve ES Cells*, Nature (2023) https://doi.org/10.1038/s41586-023-06604-5

25 Human admixed embryos are embryos created from both animal and human cells.

26 [2003] 2 WLR 692.

27 Philip Ball, *What is an Embryo? Scientists Say Definition Needs to Change*, Nature 18 August (2023).

28
*Id.*

29
Human Fertilisation and Embryology Authority, Modernising Fertility Law: Recommendations from the Human Fertilisation and Embryology Authority (HFEA) for changes to the Human Fertilisation and Embryology Act 1990 (as amended) (HFEA, 2023).

30
Jonathan Montgomery, Introduction, in Human Embryo Culture: Discussions concerning the statutory time limit for maintaining human embryos in culture in the light of some recent scientific developments (Nuffield Council on Bioethics, 2017) 3–11.

31 [2003] 2 WLR 692 at [26].

32
*Id per* Lord Bingham at [13].

33 HFEA, *supra* note 29.

34 The Governance of Stem Cell-Based Embryo Models (G-SCBEM) project is based in Cambridge and ‘brings together scientists, legal scholars and bioethics experts, as well as representatives from major funders and regulators of this research’. https://www.cam.ac.uk/research/news/project-launched-to-provide-guidance-on-research-using-human-stem-cell-based-embryo-models. Its proposals will be published in Spring 2024. See also, Amy L. Foreman et al. *Human Embryo Models: The Importance of National Policy and Governance Review,* 82 Curr. Opin. Genet. Dev. 102103 (2023).

35
Michael Mulkay, The Embryo Research Debate: Science and the Politics of Reproduction (Cambridge University Press, 1997).

36 HFEA, *supra* note 29.

37 Warnock Report, *supra* note 8, para 11.17.

38 John Finnis, *The Priority of Persons Revisited*, 58 Am. J. Jurisprud. 45–62 (2013).

39 Dan W. Brock, *Is a Consensus Possible on Stem Cell Research? Moral and Political Obstacles*,32 J. Med. Ethics 36–42 (2006); J. Koplin & J. Massie, *Lessons from Frankenstein 200 years on: Brain Organoids, Chimaeras and Other “Monsters”,* 47 J. Med. Ethics 567–571 (2021).

40 Isabel Karpin, *The Uncanny Embryos: Legal Limits to the Human and Reproduction Without Women,* 28 Sydney Law Review 599 (2006); Sarah Chan & John Harris, *Consequentialism without Consequences: Ethics and Embryo Research*, 19 Cambridge Quarterly of Healthcare Ethics 61–74 (2010); Insoo Hyun, *The Embryo Potentiality Argument Revisited: “Once More unto the Breach, Dear Friends”,* 13 Am.J. Bioeth. 28–29 (2013).

41 M. Stier & B. Schoene-Seifert, *The Argument from Potentiality in the Embryo Protection Debate: Finally “Depotentialized”?*, 13 Am.J. Bioeth. 19–27 (2013); Ana M. Pereira Daoud et al., *Modelling Human Embryogenesis: Embryo-like Structures Spark Ethical and Policy Debate*, 26 Hum. Reprod. Update 779–798 (2020).

42 Warnock Report, *supra* note 8, para 11.9. For discussion of the evidence received by the Warnock Committee, see further Natasha Hammond-Browning, *Ethics, Embryos and Evidence: A Look Back at Warnock,* 23 Med. Law Rev. 588–619 (2015).

43 Warnock Report, *supra* note 8, para 5.5.

44 See further Sarah Franklin and Emily Jackson, The 14 Day Rule and Human Embryo Research: a Sociology of Biological Translation (Routledge, 2024); Catriona McMillan, *When is Human?: Rethinking the Fourteen-Day Rule*, in The Cambridge Handbook of Health Research Regulation 365–373 (Graeme Laurie et al. eds., Cambridge University Press, 2021).

45
*Id.*

46 Mary Warnock, *Moral Thinking and Government Policy: The Warnock Committee on Human Embryology*, 63 Milbank Mem. Fund Q.: Health and Society 504(1985).

47 Franklin & Jackson, *supra* 44.

48
House of Lords Science and Technology Committee - Third Report of Session 1999-2000.

49 ISSCR, *supra* note 7; Foreman et al., *supra* note 34.

50 Mulkay, *supra* 35.

51 Franklin & Jackson *supra* note 44.

52 Stephen R. Bates et al., *“How Do We Know It’s Not Been Done Yet?!” Trust, Trust Building and Regulation in Stem Cell Research*, 37 Sci. Public Policy 703–718 (2010).

53 Sheldon Krimsky, *Ten Ways in Which He Jiankui Violated Ethics,* 37 Nat. Biotechnol. 19–20 (2019).

54 David Cyranoski, *Verdict: Hwang’s Human Stem Cells Were All Fakes,* 439 Nature 122–124 (2006).

55 Bates et al., *supra* note 52.

56
*Id.*

57
Hopkins Van Mil and HBDI, Public dialogue on research involving early human embryos, October 2023.

58 Ibid. See also Andrew J. Barnhart & Kris Dierickx, *Cultures and Cures: Neurodiversity and Brain Organoids,* 22 BMC Med. Ethics 1–6 (2021).

59 Jeremy Sugarman et al., *Critical Considerations for Public Engagement in Stem Cell-related Research,* 18 Stem Cell Rep. 420–426 (2023).

60 A. T. Clark et al., *Human Embryo Research, Stem Cell-derived Embryo models and in Vitro Gametogenesis: Considerations Leading to the Revised ISSCR Guidelines,* 16 Stem Cell Rep. 1416–1424 (2021).

61 Ana S. Iltis et al., *Ethical, Legal, Regulatory, and Policy Issues Concerning Embryoids: A Systematic Review of the Literature*, 14 Stem Cell Res. Ther. 1–15 (2023); Bernard Baertschi et al., *Organoids Research: What are the Ethical Issues?*, HAL Open Science ffinserm-03117706 (2020).

62 Juli Bollinger et al., *Patients’ Perspectives on the Derivation and Use of Organoids*, 16 Stem Cell Rep. 1874–1883 (2021). Oonagh Corrigan et al., *Ethical Legal and Social Issues in Stem Cell Research and Therapy,*Cambridge Genetics Knowledge Park 1–31 (2006).

63 Iltis et al., *supra* note 61.

64 ISSCR, *supra* note 7.

65
*Id.*

66
*Id.*

67
*Id.*

68 M. Fabbri et al., *Modeling Policy Development: Examining National Governance of Stem Cell-based Embryo Models,* 18 Regen. med. 155–168(2023).

69 In other organoid uses, the fact that an organoid will be a clone could be positively beneficial. If organoids were to be used in transplantation, the tissue donor would not need to take immunosuppressive medication, in the same way as people who have conventional organ transplants. Similarly, in the case of in vitro-derived gametes, the fact that they would be genetically identical to the skin cell donor would also be a positive advantage, because it would mean that a person without functioning gametes could have a child who was genetically related to them.

70 Patrick Pullicino, Edward J. Richard & William J. Burke, *Mass Production of Human “Embryoid” Cells from Developmentally Frozen Embryos: Is It Ethical?*, 87 Linacre Q. 347–350 (2020).

71 Para 11.5 of the Warnock Report *supra* note 8, defined the primitive streak as: ‘a heaping-up of cells at one end of the embryonic disk on the fourteenth or fifteenth day after fertilisation’. Two primitive streaks may form in a single embryonic disk. This is the latest stage at which identical twins can occur. The primitive streak is the first of several identifiable features which develop in and from the embryonic disk during the succeeding days, a period of very rapid change in the embryonic configuration. By the 17th day the neural groove appears and by the 22nd to 23rd day this has developed to become the neural folds, which in turn start to fuse and form the recognizable antecedent of the spinal cord’.

72 Franklin & Jackson, *supra* note 44.

73 Kirstin R. W. Matthews & Daniel Moralí, *National Human Embryo and Embryoid Research Policies: A Survey of 22 Top Research-intensive Countries,* 15 Regen. med. 1905–1917 (2020).

74
*Id.*

75 Monika Piotrowska, *Research Guidelines for Embryoids,* 47 J. Med. Ethics e67–e67 (2021).

76 Matthews & Morali, *supra* note 73.

77 G. Amadei et al., *Embryo Model Completes Gastrulation to Neurulation and Organogenesis*, 610 Nature 143–53 (2022).

78 Pereira Daoud et al., *supra* note 41.

79 ISSCR, *supra* note 7, para 2.2.2 g.

80 E. Christian Brugger, *The Problem of Fetal Pain and Abortion: Toward an Ethical Consensus for Appropriate Behavior*, 22 Kennedy Institute of Ethics Journal 263–287 (2012).

81 Anne McLaren, *Prelude to Embryogenesis*, in Human Embryo Research: Yes or No? The Ciba Foundation, (Cambridge University Press, 1986) 5–23. For discussion, see Sarah Franklin, *Developmental Landmarks and the Warnock Report: A Sociological Account of Biological Translation,* 61 Comp Stud Soc Hist 743–773 (2019).

82 Quoted in Michael Lockwood, *Warnock versus Powell (and Harradine): When Does Potentiality Count?,* 2 Bioethics 187–213 (1988).

83 Anne McLaren, *Where to Draw the Line,* 56 Proceedings of the Royal Institute of Great Britain 101–21 (1984).

84 Alan Holland, *A Fortnight of My Life is Missing: A Discussion of the Status of the Human ‘pre-embryo*’, 7 J. Appl. Philos. 25–37 (1990).

85 John Marshall, *The Case Against Experimentation*, in Experiments on Embryos 55–64 (Anthony Dyson & John Harris eds., Routledge, 1990); Modesto Ferrer Colomer & Luis Miguel Pastor, *The Preembryo’s Short Lifetime: The History of a Word*, 23 Cuadernos de bioética 677–694 (2012).

86 Human Fertilisation and Embryology Act 1990, Schedule 2(3A).

87 Moris et al., *supra* n 14.

88
Emily Jackson, Law and the Regulation of Medicines (Bloomsbury Publishing, 2012).

89 Posfai et al., *supra* note 22; Maxence Gaillard & Mylène Botbol-Baum, *Pursuit of perfection? On brain organoids as models*, 13 AJOB Neuroscience 79–80 (2022).

90 Ana M. Pereira Daoud, Wybo J. Dondorp & Guido M.W.R. de Wert, *The Closer the Knit, the Tighter the Fit: Conceptual and Ethical Issues of Human Embryo Modelling*, 43 Reprod. BioMed. Online 1123–1125 (2021).

91 Made more complex because the embryo itself could be described as a liminal entity, see further Catriona McMillan, The Human Embryo in Vitro: Breaking the Legal Stalemate (Cambridge University Press, 2021).

92 Rivron et al., *supra* note 11.

93 I. Hyun, *Engineering Ethics and Self-organizing Models of Human Development: Opportunities and Challenges*, 21 Cell Stem Cell 718–20 (2017).

94 Moris et al., *supra* note 14.

95 Andrew J. Barnhart & Kris Dierickx, *A RAD Approach to iBlastoids with a Moral Principle of Complexity,*Am. J. Bioeth. 2254–56 (2022).

96 Human Fertilisation and Embryology Act 1990 Schedule 3(2)(c).

97
Modernising fertility law: Recommendations from the Human Fertilisation and Embryology Authority for changes to the Human Fertilisation and Embryology Act 1990, as amended (HFEA, 2023).

98 Sarah Franklin, *Embryonic Economies: The Double Reproductive Value of Stem Cells,* 1 BioSocieties 71–90 (2006).

99 Julian J. Koplin, *Response to the ISSCR Guidelines on Human–animal Chimera Research*, 37 Bioethics 192–198 (2022).

100 Criminal sanctions are also common across Europe, see further Rosario Isasi et al., *Mending the Gaps: Ethically Sensitive Cells and the Evolution of European stem Cell Policy,* 17 Regen. Med. 581–595 (2022).

101 Human Fertilisation and Embryology Authority 1990, Schedule 2(3)(5).

102 Moris et al., *supra* note 14.

103 W. M. S. Russell and R. L. Burch, The Principles of Humane Experimental Technique (Methuen & Co, 1959).

104 Moris et al., *supra* note 14. See also Pereira Daoud et al., *supra* note 90.

105 HFEA, *supra* note 29.

106 Sarah Chan, *How and Why to Replace the 14-day Rule*, 4 Curr. Stem Cell Rep. 228–234 (2018).

107 Paola Nicolas, Fred Etoc & Ali H. Brivanlou, *The Ethics of Human-embryoids Model: A Call for Consistency*, 99 J. Mol. Med. 569–579 (2021).

108 Kathy Niakin, ‘Keeping Our Promise to Patients: Ensuring that We Learn from Donated Embryos’ paper delivered at Progress Educational Trust Conference 6 Dec. 2023.

109
Sarah Franklin et al., Factors affecting PGD patients’ consent to donate embryos to stem cell research, Paper presented at the Sixth International Symposium on Preimplantation Genetics, London, 19–21 May 2005. (See Conference Programme and Abstracts, Reproductive BioMedicine Online, 10, Suppl. 2, 31.); Catarina Samorinha et al., *Factors Associated with the Donation and Non-donation of Embryos for Research: A Systematic Review,* 20 Hum. Reprod. Update 641–655 (2014).

110 Patrick Pullicino, Edward J. Richard & William J. Burke, *Mass Production of Human “Embryoid” Cells from Developmentally Frozen Embryos: Is It Ethical?*, 87 Linacre Q. 347–350 (2020).

111 Kevin Wilger, *Embryo Models Derived from Stem Cells: A Response to Nicolas Rivron and Colleagues*, 19 Nati. Cathol. Bioeth. Q. (2019); Kevin Wilger, *Gaps in Embryo Model Ethics*, 45 Ethics Med. 1–4 (2020).

112 Moris et al., *supra* note 14.

113 I am grateful to Julian Koplin for this point.

114 Hinterberger & Bea, *supra* note 20.

115
Agence de la Biomédicine, Opinion of the Conseil d’Orientation: Stem Cell-Based Embryo Models (2023).

116 Foreman et al., *supra* note 34; Rachel A. Ankeny, Megan J. Munsie & Joan Leach, *Developing a Reflexive, Anticipatory, and Deliberative Approach to Unanticipated Discoveries: Ethical Lessons from iBlastoids,* 22 Am. J. Bioeth. 36–45 (2022).

117 Franklin & Jackson, *supra* note 44.

